# Incorporating Spatial and Spectral Saturation Modules Into MR Fingerprinting

**DOI:** 10.1002/nbm.70000

**Published:** 2025-01-25

**Authors:** Christopher G. Trimble, Kaia I. Sørland, Chia‐Yin Wu, Max H. C. van Riel, Tone F. Bathen, Mattijs Elschot, Martijn A. Cloos

**Affiliations:** ^1^ Department of Circulation and Medical Imaging Norwegian University of Science and Technology Trondheim Norway; ^2^ Department of Radiology and Nuclear Medicine St. Olavs Hospital, Trondheim University Hospital Trondheim Norway; ^3^ Centre for Advanced Imaging The University of Queensland St Lucia Queensland Australia; ^4^ ARC Training Centre for Innovation on Biomedical Imaging Technology (CIBIT) The University of Queensland St Lucia Queensland Australia; ^5^ School of Electrical Engineering and Computer Science The University of Queensland St Lucia Queensland Australia; ^6^ Computational Imaging Group for MR Diagnostics and Therapy, Department of Radiotherapy UMC Utrecht Utrecht The Netherlands; ^7^ Donders Centre for Cognitive Neuroimaging, Donders Institute for Brain, Cognition and Behaviour 12 Radboud University Nijmegen Netherlands

**Keywords:** artefacts, extended phase graph, MR fingerprinting, prostate, radial MRI, saturation

## Abstract

In this work, we introduce spatial and chemical saturation options for artefact reduction in magnetic resonance fingerprinting (MRF) and assess their impact on *T*_1_ and *T*_2_ mapping accuracy. An existing radial MRF pulse sequence was modified to enable spatial and chemical saturation. Phantom experiments were performed to demonstrate flow artefact reduction and evaluate the accuracy of the *T*_1_ and *T*_2_ maps. As an in vivo demonstration, MRF of the prostate was performed on an asymptomatic volunteer using saturation modules to reduce flow‐related artefacts. *T*_1_, *T*_2_ and *B*_1_^+^ maps obtained with and without saturation modules were compared. Application of spatial saturation in prostate MRF reduced streaking artefacts from the femoral vessels. When saturation is enabled *T*_1_ accuracy is preserved, and *T*_2_ accuracy remains acceptable up to approximately 100 ms. Chemical and spatial saturation can be incorporated into MRF sequences with limited impact on *T*_1_ accuracy. Further sequence optimisation may be needed to accurately estimate long *T*_2_ components. Spatial saturation modules have potential in prostate MRF applications as a means to reduce flow‐related artefacts.

## Graphical abstract

A radial MR Fingerprinting sequence is modified to include fat and spatial saturation options. We demonstrate in phantoms and in volunteers the reduction of flow‐related streak‐like artefacts while *T*_1_ accuracy is well preserved and *T*_2_ measurements remain acceptable up to approximately 100 ms.

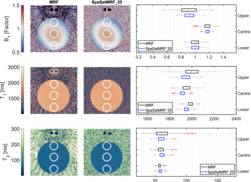

**Abbreviations**
ADC: apparent diffusion coefficient
CRLB: Cramér–Rao lower bound
EPG: extended phase graph
FISP: fast imaging with steady‐state precession
FLASH: fast low angle shot
mp‐MRI: multiparametric MRI
MRF: magnetic resonance fingerprinting
RF: radio frequency
ROI: region of interest
ROVir: region‐optimised virtual
SatMRF: saturation‐enabled MRF
SNR: signal‐to‐noise ratio
SVD: singular value decomposition

## 1 Introduction

Saturation modules are a ubiquitous tool in MRI. Specifically, spatial and chemical saturation techniques have brought invaluable capability to clinical imaging [1]. Chemical saturation modules are commonly used to avoid chemical shift artefacts and eliminate bright fat signals in *T*_1_‐weighted images [2, 3]. Spatial saturation modules can enhance image quality by suppressing signals from areas prone to induce artefacts through respiratory motion or fluid flow [4, 5]. Although prevalent in traditional imaging sequences, spatial saturation modules have not yet been demonstrated in MR fingerprinting (MRF). Chemical saturation has been demonstrated in MRF however, with several recent works employing fat saturation to enhance contrast and reduce artefacts, either as a preparation pulse or within every TR [6, 7, 8, 9], but not yet periodically interwoven within the variable flip angle train.

Since the landmark MRF paper in 2013 [10], there have been numerous developments of the technique [11]. Optimisation of the flip angle train in combination with improved sampling strategies and reconstruction techniques has enabled faster imaging with higher resolutions [12, 13]. Other works have explored the ability to map a variety of tissue properties [14, 15, 16, 17, 18, 19, 20, 21] and mitigate experimental imperfections [22, 23, 24, 25]. Nevertheless, clinical application of MRF is not always trivial because of challenges in standardisation and validation but also prevalent inflow or motion artefacts. The application of saturation modules to MRF in abdominal exams, such as for prostate cancer diagnosis, may help reduce such image artefacts.

In general, the benefit of quantitative MRI is the robust measurement of physical properties as a biomarker of healthy or diseased tissue [26]. Quantitative MRI enables the comparison of such measurements between patients, scanners and time points. Take prostate cancer diagnosis as an example, where ADC is strongly correlated to Gleason score, a measure of tumour aggressiveness [27]. Currently, multiparametric MRI (mpMRI) protocols are often used, where image interpretation according to the Prostate Imaging and Data Reporting System (PI‐RADS) guidelines predominantly relies on subjective analysis of mpMRI findings, incorporating very few quantitative features [28]. Typically, the mpMRI protocol contains *T*_2_‐weighted imaging; diffusion‐weighted imaging, including apparent diffusion coefficient (ADC) maps and high *b*‐value images; and dynamic contrast enhanced (DCE) imaging [29, 30, 31].

MRF presents an opportunity to build upon the parametric mapping of the prostate for improved cancer detection and classification. Previous works have shown that simultaneous *T*_1_ and *T*_2_ MRF maps combined with conventional ADC mapping can aid the differentiation between prostate tissue and cancerous lesions [32, 33, 34, 35, 36, 37]. However, MRF of the prostate comes with some challenges. Blood flowing through the femoral arteries and veins produces bright signals with an unstable phase. When using radial sampling, these vessels produce streak‐like artefacts that deteriorate the precision of the tissue maps [38]. Similarly, strong signals from subcutaneous fat close to the receiver coils are known to produce image artefacts in radial imaging [7, 39]. In addition, inhomogeneities in the *B*_0_ and *B*_1_^+^ field can impact the accuracy [40, 41, 42].

The aim of the current work was to introduce saturation modules into the MRF framework and evaluate the modified sequences against the baseline MRF. We modified an existing radial MRF pulse sequence [43] chosen for its inherent *B*_1_^+^ insensitivity and good reproducibility in terms of *T*_1_ and *T*_2_ estimation. The sequence has previously been evaluated in phantoms against spin echo and inversion recovery spin echo (IRSE) methods [22].

The modified sequence was validated with phantom experiments as a proof of principle. Demonstrating a potential clinical application, we then performed MRF of the prostate in five asymptomatic volunteers with spatial and chemical saturation. Multiparametric (*T*_1_, *T*_2_ and *B*_1_^+^) maps obtained with and without saturation modules were compared.

## 2 Theory

Saturation modules utilise a 90° radiofrequency (RF) pulse followed by a spoiling gradient to suppress either a chemically or spatially isolated fraction of the magnetisation [5, 44, 45]. Fat saturation, for example, uses an RF pulse centred on the fat frequency. Provided that the pulse bandwidth does not overlap with the water peak, only the fat signal is supressed. Spatial saturation modules use slice‐selective RF pulses to locally saturate magnetisation, such that these areas contribute minimal signal to the image. However, care must be taken not to deteriorate the intended image contrast when incorporating saturation modules in existing sequences.

MRF sequences offer a rich design space because they do not operate in the steady state. Instead, the dynamics observed in transient MRI signals are used to infer tissue properties and even experimental factors. Nevertheless, some transients have higher encoding power than others [46]. To quantify *T*_2_ signal, contributions must be refocused multiple times.

In this work, we modify an MR Fingerprinting sequence to include saturation modules. The following section describes how spatial and spectral saturation modules can impact the signal evolution in MRF sequences. In particular, the gradient moments in the saturation modules must be tuned such that the desired coherence pathways are still refocused at the right time. The extended phase graph (EPG) formulism can be used to study and visualise the refocusing of these coherence pathways [47].

**FIGURE 1:**
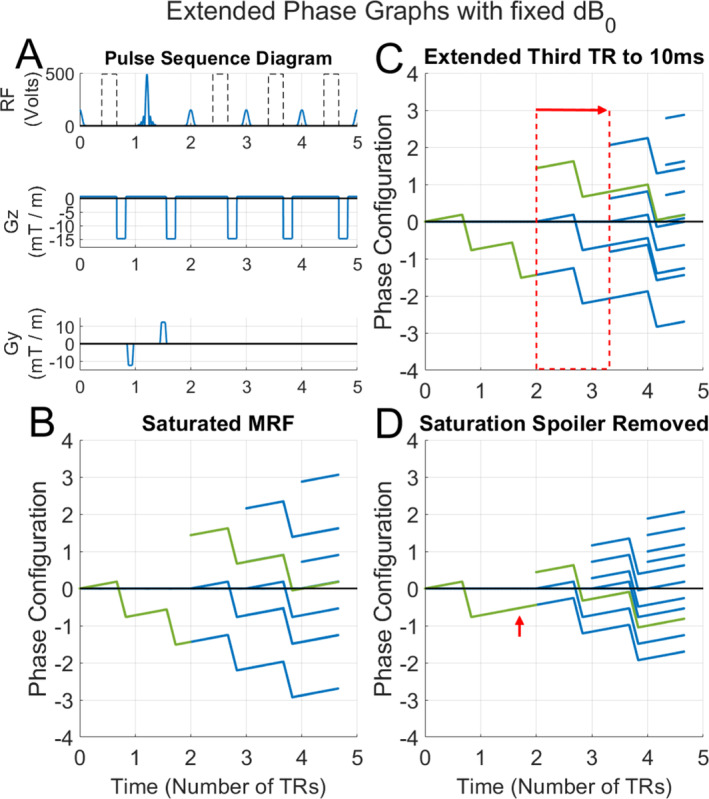
Pulse sequence diagram and associated EPGs showing the phase configuration state with respect to the slice direction against time. (A) A simplified MRF Pulse Sequence Diagram of a FISP sequence with a 1‐TR saturation block. Dotted lines show the signal readouts. The saturation occurs during the second TR. Gradient axes Gz and Gy represent the total gradient momentum applied in the slice and phase direction respectively. In FISP, the phase gradient is balanced; the two gradients shown here highlight the balanced gradients played before and after the saturation RF pulse. (B) Extended phase graph with fixed background ∆ *dB*_0_ in the slice direction. Each blue line represents a possible phase evolution produced by the pulse sequence; the green line highlights an example evolution, which is correctly refocused in the fifth TR. (C) As B but the third TR is extended in duration (red box and arrow), resulting in a loss of coherence. (D) As B, but without spoiling after the saturation pulse, which fails to refocus the signal generated prior to saturation, as represented by the green line. The red arrow shows where the spoiling gradient would have been placed.

Many MRF sequences leverage concepts from inversion recovery fast imaging with steady‐state precession (FISP) to encode both *T*_1_ and *T*_2_ information. A segment of a FISP pulse sequence diagram including a 1‐TR saturation module is shown in Figure 1A. The gradients in the slice (Gz) and phase (Gy) direction are simplified for visual clarity. The corresponding EPG shows the evolution of coherence pathways (Figure 1B) where the phase configuration state shown refers to the transversal phase configuration state (*F*‐state) rather than the longitudinal (*Z*‐state). Signal is optimally acquired when the *F*‐state reaches zero as the signal is then in the transverse plane.

Each RF excitation generates one new pathway beginning at phase configuration state zero. The spoiling gradient provides a *2n* × *π* phase twist across the voxel, where *n* is an integer, shifting all coherence pathways by one phase configuration step. In addition to exciting new signal, RF excitations also have a refocusing component, flipping the sign of their phase state. Because of this refocusing component, subsequent gradient moments will return the pathway back to phase state configuration zero and thus contribute to acquired signal.

Figure 1B also shows the evolution of coherence pathways in an FISP segment when a saturation module is added with a duration and effective spoiling moment equal to those in the imaging modules. In addition, these simulations include some phase accumulation due to field inhomogeneities (*dB*_0_). Such inhomogeneities are known to cause inaccuracy in quantitative measurements and an important consideration for sequence design [42, 48, 49]. For comparison, Figure 1C,D shows the distortions in the evolution of coherence pathways in a FISP segment due to variable TR length and a mismatch in spoiling moment, respectively. In both of these examples, incomplete refocusing due to *dB*_0_ results in loss of coherence and thus a loss in *T*_2_ measurement accuracy. Figure S1 provides the pulse sequence for each phase graph shown in Figure 1.

When considering the signals we intend to suppress, the aim is to avoid phase configuration state zero at the time of signal readout. To achieve this, the saturation RF pulse is placed in between a pair of balanced spoiling gradients played on the read and phase axis (Figure 1A). These gradients, in combination with the unbalanced spoiling moment of the slice direction effectively prevent the saturated spins from reaching the zero‐phase state.

**FIGURE 2:**
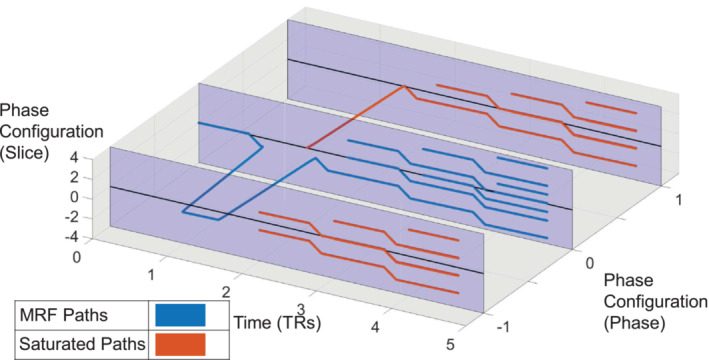
3D extended phase graph of a saturation‐equipped MRF sequence (as shown in Figure 1A) with fixed background *dB*_0_ in the slice direction. The paths in blue represent the phase evolutions of excited signal while the red lines show the phase evolution of the saturated signal. The saturated signal receives a phase configuration in the phase direction of the voxel, which prevents saturated signal from contributing to the acquired signal.

To visualise the saturated pathways, Figure 2 plots the EPG of the sequence described in Figure 1A, with transversal phase configuration states in the slice and phase directions. Here, the signal generated from the saturation pulse is subject to an unbalanced gradient in the phase direction. Meanwhile, the signal generated prior to the saturation pulse experiences a net‐zero gradient moment in the phase direction and thus maintains a zero‐phase state configuration in that direction. Saturated pathways are refocused by subsequent RF pulses and may achieve configuration state zero in the slice direction but never in the phase direction. Therefore, the magnetisation targeted by the saturation pulse no longer contributes to the acquired signal in this sequence.

## 3 Methods

### 3.1 Sequence Design

**FIGURE 3:**
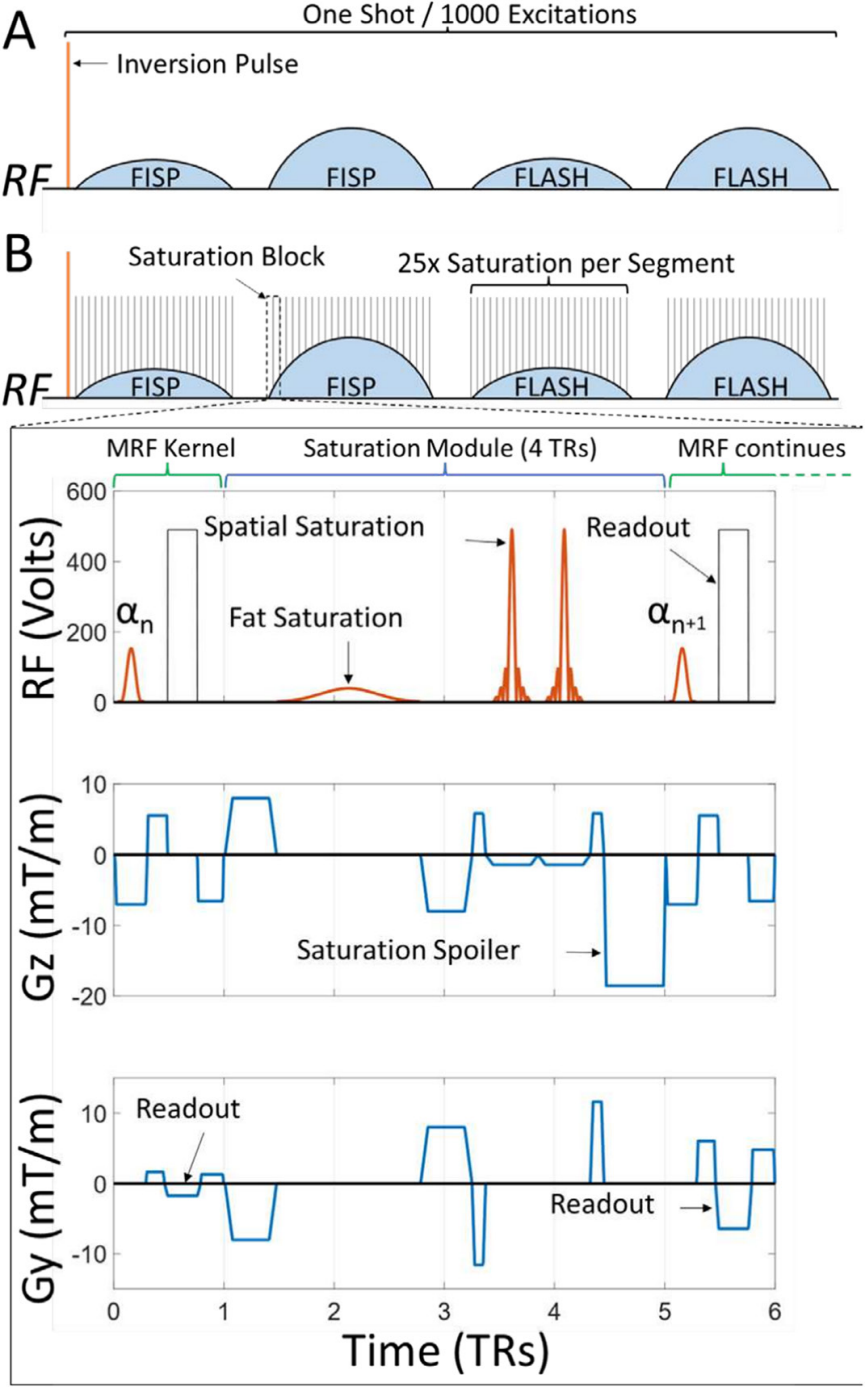
(A) Flip angle train for the MR Fingerprinting pulse sequence. (B) Pulse sequence diagram of AllSatMRF_10 sequence with both chemical (fat) and two spatial saturation pulses. On the RF axis, the red lines show the RF pulses, whereas the black rectangles indicate the analogue‐to‐digital converter (ADC) readout events. The Gz axis shows the slice gradients in blue including saturation spoiling gradient. Gy axis shows balanced readout and saturation gradients.

We added saturation modules to the radial MRF pulse sequence described by Cloos et al. [43]. The sequence begins with a non‐selective adiabatic inversion pulse and is followed by 1000 RF excitations divided into four segments: two FISP and two fast low angle shot (FLASH) (Figure 3A). Both the FISP and FLASH segments had 8π gradient spoiling. In addition to the gradient spoiling, the FLASH segments also had 50° quadratic phase spoiling. The TR/TE was fixed at 7.5/3.5 ms, whereas the nominal flip angle varied between 3.2° and 60.0°. Between segments there was a 50‐TR break, this was part of the MRF implementation as detailed in Cloos et al. [43] to allow for some additional *T*_1_ recovery and *T*_2_ decay between segments. The set of radial readout angles depending on the number of shots (*n*_l_) selected and can be defined as $\alpha =\mathrm{mod}\left(2\pi ,\theta \cdot \left(s\cdot n_{l}+l\right)+\pi \delta_{0,\mathrm{mod}\left(s.2\right)}\right)$, where s is the readout index, l is the shot index, mod the modulo operator, *θ* is the golden angle (111.25°) and δ is the Kronecker delta function [50].

Saturation was incorporated into the MRF sequence in several different configurations, as a general term we will refer to saturation‐enabled MRF as SatMRF. Initially, two spatial saturation pulses were added in 2‐TR blocks played every 25th TR. We call this sequence SpaSatMRF_25. The default sequence building block (SBB) was applied enabling saturation bands of up to 150 mm. This limit could be increased if required; however, larger spatial saturation results in a reduction in slice profile sharpness. Conversely, thinner saturation pulses require more time to accommodate the requisite RF pulse.

To analyse the impact of saturation frequency, we made another sequence with saturation every tenth TR: SpaSatMRF_10. To accommodate chemical saturation pulses and gradients, the duration of the saturation block was necessarily increased to 4TR, and we retained a saturation frequency of every tenth TR to overcome the short *T*_1_ of fat. Finally, the AllSatMRF_10 sequence allows for both chemical saturations and two spatial saturation bands within the 4‐TR saturation block.

Table 1 provides an overview of the key sequence design parameters used in these sequences. It should be noted that each of these sequences had dedicated dictionaries, except for FatSatMRF_10 and AllSatMRF_10, which shared the same dictionary as the different saturation pulses are assumed to have no effect on the spin dynamics.

**TABLE 1:** Experimental scanning protocol, MRF and all combinations of fat and spatial saturation with SatMRF.

Sequence name	Saturation duration (TR)	Time between saturations (TR)	Spatial saturation (2×)	Chemical (fat) saturation
MRF	N/A	None	None	None
SpaSatMRF_25	2	25	Yes	No
SpaSatMRF_10	2	10	Yes	No
FatSatMRF_10	4	10	No	Yes
AllSatMRF_10	4	10	Yes	Yes

Figure 3 provides a detailed description of the original MRF sequence and the modified AllSatMRF_10 as an example. In the modified sequence, saturation modules were introduced, briefly interrupting the flip angle train every 10th TR. As explained in Section 2, the total momentum in each gradient direction of the saturation modules matches that of 4‐TR cycles of the original MRF sequence.

### 3.2 Cramér–Rao Lower Bound (CRLB)

The encoding capabilities of the different sequences were compared using the CRLB. The CRLB gives a lower bound on the variance of any unbiased estimator, in this case, the dictionary matching step of the MRF reconstruction. Thus, when a sequence has a lower CRLB value for a certain parameter, it indicates a higher encoding sensitivity for that parameter.

The MRF reconstruction is formulated as a parameter estimation problem, where we want to estimate the values of the parameter vector $\theta ={\left[T_{1},T_{2},B_{1}^{+},\rho \right]}^{T}$ from the measured signal evolutions. Here, ρ is included as a nuisance parameter that includes any scaling factor due to proton density and receive sensitivities. According to the CRLB, the variance of any parameter estimate ${\hat{\theta }}_{i}$ has the following lower bound:

$$\mathrm{Var}\left({\hat{\theta }}_{i}\right)\geq {\left[I^{-1}\left(\theta \right)\right]}_{i,i},$$

where $I\left(\theta \right)$ is the Fisher information matrix (FIM) and ${\left[\cdot \right]}_{i,i}$ is the ith diagonal value of a matrix. Assuming white Gaussian noise, the FIM can be calculated as

$$I\left(\theta \right)=\frac{1}{\sigma^{2}}\sum_{n=1}^{N}J_{n}^{T}\left(\theta \right)J_{n}\left(\theta \right).$$

The vector $J_{n}\left(\theta \right)$ is the Jacobian of $m_{n}$, the fingerprint signal after the nth excitation, that is, $J_{n}\left(\theta \right)=\left[\frac{\partial m_{n}}{\partial T_{1}},\frac{\partial m_{n}}{\partial T_{2}},\frac{\partial m_{n}}{\partial B_{1}^{+}},\frac{\partial m_{n}}{\partial \rho }\right]$, and σ is the noise level.

The Jacobian matrices were obtained from finite differences using the same simulation code that generated the dictionary. Because the noise level only scales the final values, it was set to 1. Finally, the CRLB values were normalised to remove large‐scale differences between the parameters:

$$\text{nCRLB}\left(\theta_{i}\right)=\frac{\sqrt{\text{CRLB}\left(\theta_{i}\right)}}{\theta_{i}}.$$

### 3.3 Flow Phantom

In order to evaluate the efficacy of the SatMRF sequences in artefact reduction, we conducted a flow phantom experiment. The flow phantom consisted of a cylindrical container filled with 3% agar and 2.5‐g/L sodium chloride, chosen to avoid bulk motion or flow within the flow phantom. Two tubes were then placed close to the flow phantom, with a pump supplying a 1‐Hz pulsatile flow of water through the tubes. The flow phantom was scanned on a Siemens (Siemens Healthineers, Erlangen, Germany) Prisma 3T MRI system with 18 channels from the flex‐body‐array and spine‐array coils. Foam padding was inserted for stability. Fingerprinting sequences were acquired with 1.15 × 1.15 mm resolution, 5 mm slice thickness and 240 × 240 mm field of view and one shot, which was repeated four times such that averages could be made to increase the signal‐to‐noise ratio (SNR).

### 3.4 Multicompartment Phantom

To assess the effect of the sequence modifications on *T*_1_ and *T*_2_ quantification, we scanned a custom‐made multicompartment phantom. The multicompartment phantom, based on the design from Cloos et al. [43], held ten 55‐mL cylinders containing a range of manganese (II) chloride concentrations and two 15‐mL containers containing peanut oil. A 3D‐printed insert held the samples upright within a plastic container filled with a sodium chloride solution (3 g/L).

The multicompartment phantom was scanned on a Siemens (Siemens Healthineers, Erlangen, Germany) Skyra 3T MRI system with a 32‐channel head coil with foam padding inserted for stability. The in‐plane resolution was 1 × 1 mm using a 240 × 240 mm field of view and a 5 mm slice thickness and four shots. Two slices were acquired with a slice gap of 800% to reduce crosstalk. Two spatial saturation bands with 50‐mm thickness were placed outside the field of view. The multicompartment phantom was allowed to rest at isocentre for 30 min before scanning to minimise residual bulk fluid motion.

It should be noted that the flow and multicompartment phantoms were developed at separate facilities and were therefore scanned on different scanners and at slightly different resolutions.

### 3.5 In Vivo Experiments

To explore a potential clinical application of the SatMRF sequence, we scanned five asymptomatic volunteers using a Siemens Skyra 3T MRI system (Siemens Healthineers, Erlangen, Germany) with a flexible body coil and in‐built spinal coils. The image resolution was 1.15 × 1.15 mm using a 240 × 240 mm field of view and a 5‐mm slice thickness and four shots. The prostate was imaged with two transversal slices separated by a 50% slice gap.

**FIGURE 4:**
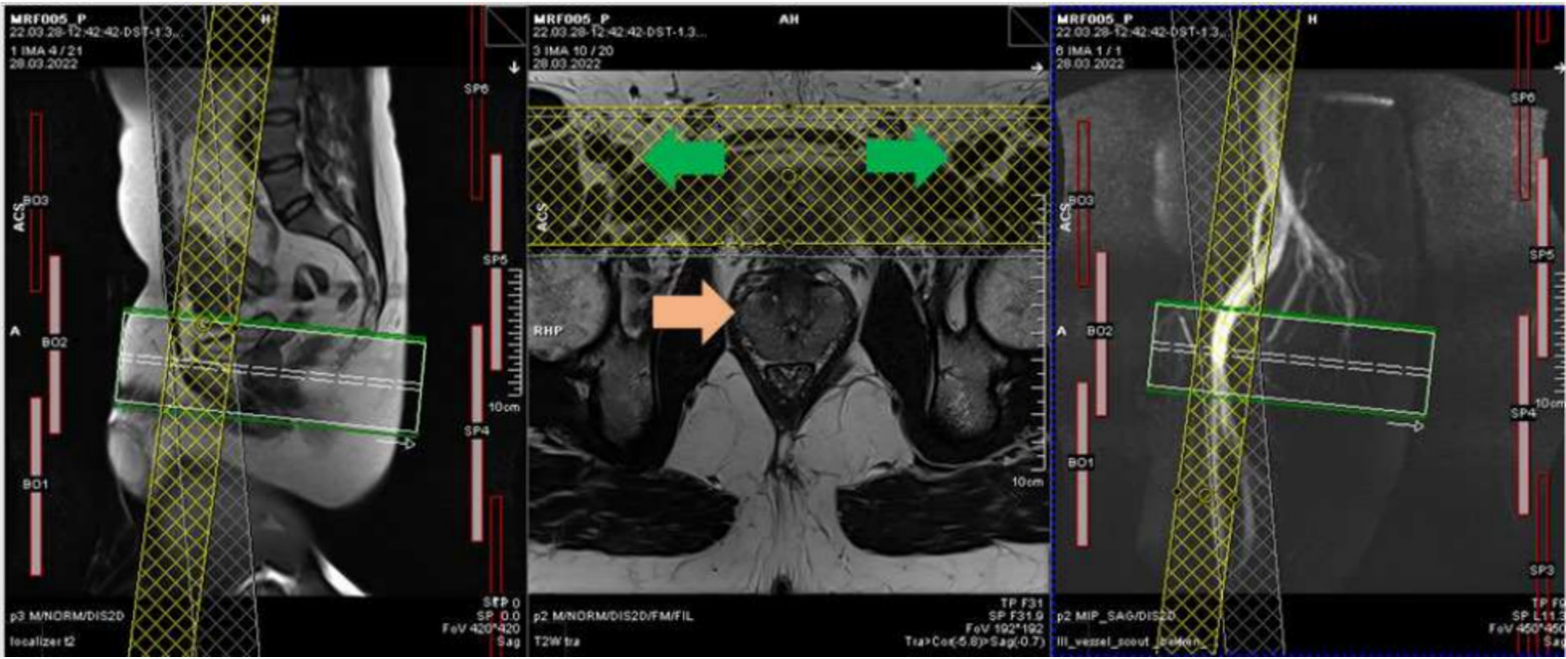
Screenshot from the MRI scanning computer with sagittal localiser (left), *T*_2_ *w* transversal (centre) and sagittal vessel scout scan (right). The yellow and grey cross‐hatched boxes show an example of how the saturation bands were oriented to supress signal from the vessels and hence reduce artefacts. On the *T*_2_ *w* image, the femoral vessels are dark and circular, shown with green arrows. The prostate is indicated with the light orange arrow.

A peripheral vessel scout scan, typically used in MR angiography, was performed to guide the positioning of the saturation bands (Figure 4), with the aim to cover as much of the femoral and lower iliac vessels as possible. The same combinations of spatial and fat saturation were tested as in the phantom study (Table 1).

The Regional Committee for Medical and Health Research Ethics (REC Central Norway) approved the study, and all the asymptomatic volunteers signed informed consents prior to recruitment (REC identifier #218665).

### 3.6 Dictionary Simulation and Image Reconstruction

The dictionaries were simulated based on EPG theory with 140 *T*_1_ entries [140–4642 ms] and 160 *T*_2_ entries [10–507.1 ms] both with 2.5% incremental step size. *B*_1_^+^ values range from 1 to 89 in 1° increments. Dictionary simulations included the slice profile as previously detailed in Cloos et al. [43] and Ma et al. [10]. Separate dictionaries were generated for each of the sequence variations as described in Table 1. The dictionaries for each of the sequences were compressed in the time domain using singular value decomposition (SVD) with a rank of 8 [46]. The time‐bandwidth product was 3, and the number of slice bins was set to 32.

All phantom and volunteer raw data (.dat files) were exported for offline reconstruction using MATLAB (MathWorks, Natick, MA). The dictionary compression was applied to the acquired data (in k‐space). Images were reconstructed using the non‐linear Fourier Transform tools provided by Fessler et al. [51]. Coil data were combined using a matched filter combination. The matching algorithm then searched the dictionary for the best match (highest correlation) on a voxel‐by‐voxel basis. The final *T*_1_, *T*_2_ and *B*_1_^+^ maps were exported using the NifTI format for segmentation and analysis. The *B*_1_^+^ maps shown in this work were normalised with a nominal flip angle of 60°. *T*_1_ and *T*_2_ maps in this paper utilise the Lipari and Navia colour maps as recommended by the Quantitative MR Study Group of the International Society of Magnetic Resonance in Medicine (ISMRM) [52].

### 3.7 Data Analysis

Image segmentation was done with ITK‐SNAP [53]. To analyse the multicompartment phantom, data circular masks of equal size (25.3‐mm diameter) were manually placed on each of the manganese (II) chloride solution samples. Smaller circular masks (13.8‐mm diameter) were placed on the peanut oil samples. For the in vivo analysis, the prostate was segmented manually by a researcher (C.G.T.) with 2 years of experience in prostate MRI. Motion between scans was assumed to be negligible. Therefore, the same segmentation mask was applied to all maps (*T*_1_, *T*_2_, *B*_1_^+^) from all acquisitions (Table 1).

To assess the differences in *T*_1_ and *T*_2_ measurements, the mean values of the segmented samples were calculated and plotted (MRF vs. SatMRF). To improve data visualisation, the relative difference to the original MRF sequence was calculated for each sequence and plotted against the mean MRF measurement per sample.

## 4 Results

As an initial evaluation of the SatMRF sequences, the CRLB was calculated for a range of *T*_2_ values around the approximate *T*_1_ of prostate tissue (Table 2). In almost all cases, the saturation‐equipped sequence performed worse than MRF for *T*_1_ and *T*_2_. The CRLB method might be applied in further work to optimise the SatMRF sequence.

**TABLE 2:** Normalised Cramér–Rao lower bound (CRLB) values calculated for three *T*_2_ values; 30, 60 and 120 ms for a fixed *T*_1_ of 1500 ms and *B*_1_^+^ of 60°. Lower values indicate a lower variance in the parameter estimation and therefore better encoding capabilities of the sequence for the given parameter.

	*T*_1_ = 1500 ms, *B*_1_ = 60 deg											
	*T*_2_ = 30 ms				*T*_2_ = 60 ms				*T*_2_ = 120 ms			
	CRLB *T*_1_	CRLB *T*_2_	CRLB *B*_1_^+^	CRL*B* ρ	CRLB *T*_1_	CRLB *T*_2_	CRLB *B*_1_^+^	CRLB ρ	CRLB *T*_1_	CRLB *T*_2_	CRLB *B*_1_^+^	CRLB ρ
MRF	3.27	10.41	4.20	3.25	2.90	7.74	3.65	2.70	2.66	7.46	3.26	2.59
SpaSatMRF_25	3.33	12.49	4.16	3.36	3.01	10.77	3.68	2.75	2.81	11.92	3.39	2.62
SpaSatMRF_10	3.41	15.35	4.09	3.54	3.12	12.95	3.76	2.77	2.96	10.57	3.56	2.53
FatSatMRF_10	3.45	13.84	3.86	3.40	3.18	12.03	3.54	2.71	3.02	9.89	3.37	2.43

### 4.1 Flow Phantom

**FIGURE 5:**
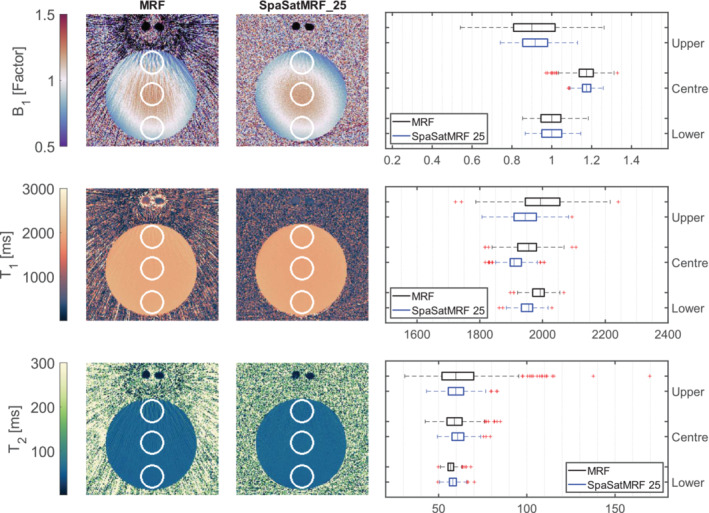
A spherical (3% agar, 2.5 g NaCl/L) flow phantom was scanned with MRF, and SpaSatMRF_25 sequences with two tubes of flowing water were placed near the flow phantom. Here, we show the *B*_1_^+^, *T*_1_ and *T*_2_ maps, generated from MRF and SpaSatMRF_25. SatMRF data were acquired with one spatial saturation band covering the flowing water tubes. The SpaSatMRF_25 sequence visibly reduces streak artefacts in all maps. Box plots of MRF maps are shown where three circular ROIs were placed at the upper, centre and lower positions of the flow phantom. The upper ROI contains the most streak artefacts, and in each of the *T*_1_, *T*_2_ and *B*_1_^+^ maps, the standard deviation of values within the ROI is decreased when using the saturated enabled MRF sequence.

Next, we evaluate our phantom data; Figure 5 demonstrates the effect of flow on the unmodified MRF sequence with *T*_1_, *T*_2_ and *B*_1_^+^ maps of the flow phantom. Strong streak‐like artefacts radiate from the tubes filled with flowing water. When a spatial saturation band was placed over the tubes, the flow artefacts in the *T*_1_ and *T*_2_ maps were removed almost completely. Some residual streak artefacts remained in the *B*_1_^+^ map but are markedly reduced compared with the original MRF maps. The three regions of interest (ROIs) in the maps correspond to the box plots on the right side of Figure 5. For the upper ROI, where streaking is most intense, the standard deviation of values in the saturated MRF maps is much smaller. For the central and lower ROIs, there is little difference, as streak intensity reduced with distance from the tubes.

### 4.2 Multicompartment Phantom

**FIGURE 6:**
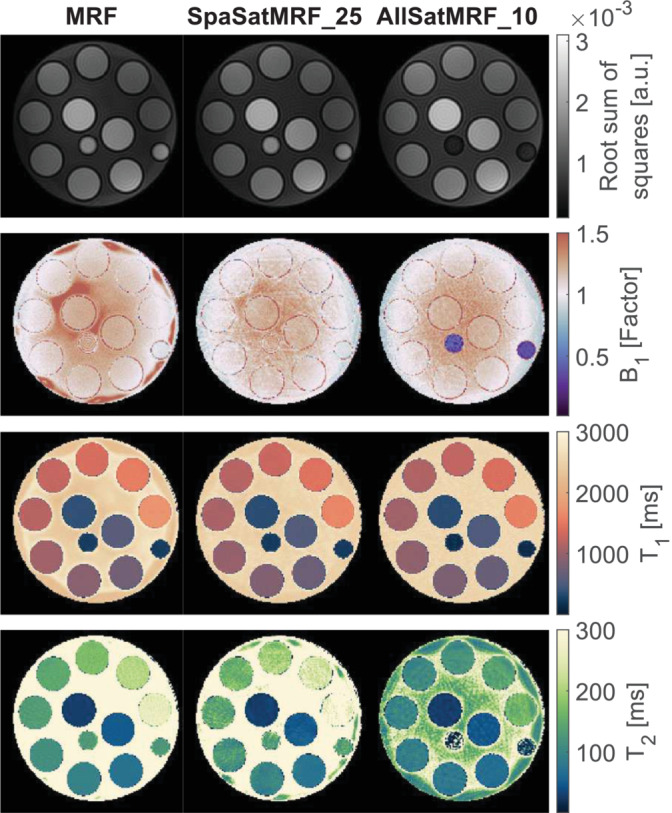
Top row shows the root‐sum‐of‐squares combination of all coil images in the scan. Next are the *B*_1_^+^, *T*_1_ and *T*_2_ maps, generated from MRF, SpaSatMRF_25 and AllSatMRF_10. Our custom‐built multicompartment phantom is composed of 10 test tubes each containing a different concentration of paramagnetic manganese chloride solution, designed to span a range of *T*_1_ and *T*_2_ values. The smaller cylinders containing peanut oil are placed at the centre and the periphery of the multicompartment phantom to simulate visceral and subcutaneous fat respectively.

Multiparametric maps generated using MRF and SatMRF sequences are shown in Figure 6. Additionally, the root sum of squares combined coils images using all k‐space data are given. This image demonstrates that signal from fat samples was suppressed with the enabled fat saturation module. After suppression, the residual signal in the oil samples best matched dictionary entries with a low *B*_1_^+^, long *T*_1_ and *T*_2_. Outside the oil samples, *B*_1_^+^ maps obtained with all three methods appear similar. *T*_1_ maps show little variation between sequences, although bulk fluid in the multicompartment phantom appears to be overestimated in SpaSatMRF_25. The *T*_2_ maps from the SatMRF sequence show increased artefact distortion compared with the MRF maps. Two of the compartments have *T*_2_ values above 200 ms, which are clearly underestimated in the AllSatMRF map.

**FIGURE 7:**
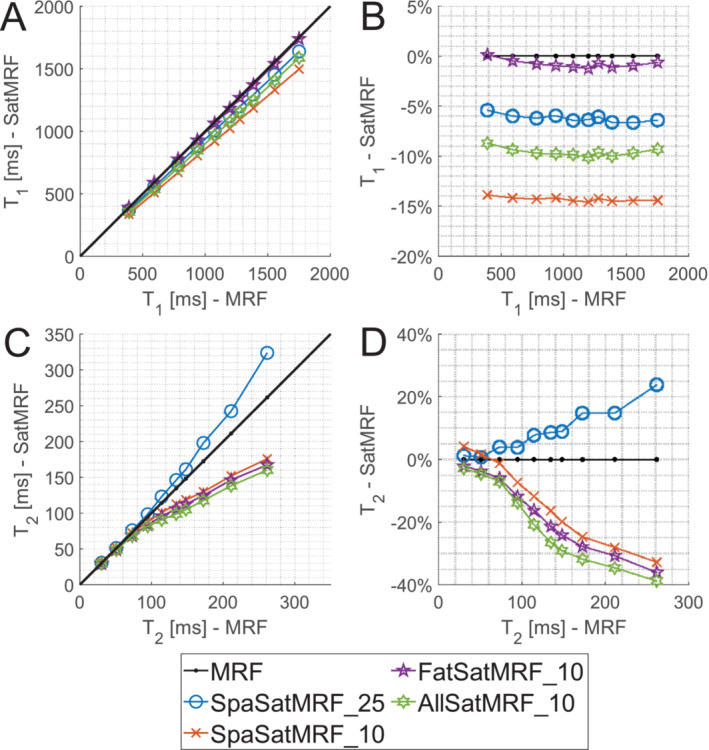
Plot of *T*_1_ (A,B) and *T*_2_ (C,D) measurements of the multicompartment phantom. Mean values (A,C) of each segmented sample as measured by MRF, against respective SatMRF measurements. Relative difference (B,D) of SatMRF measurements using different saturations versus MRF.

Figure 7 plots the mean *T*_1_ and *T*_2_ values of the multicompartment phantom when using each of the MRF sequences given in Table 1. The percentage difference of SatMRF sequences *T*_1_ relative to MRF *T*_1_ is given in Figure 7B. SpaSatMRF_25 underestimates *T*_1_ by approximately 5%–6%. Increasing the frequency of saturation appears to reduce *T*_1_ accuracy with SpaSatMRF_10 measuring *T*_1_ at 14%–15% less than MRF; however, AllSatMRF_10 measures *T*_1_ at around 10% below MRF. The best performing sequence with respect to *T*_1_ was FatSatMRF_10 with only fat saturation enabled; here, *T*_1_ measurements were within 1.5% of MRF values.

The *T*_2_ measurement accuracy of SatMRF decreases with increasing *T*_2_ value (Figure 7D). SatMRF *T*_2_ values up to 95 ms were within 4% of the MRF measurement. Up to 50 ms, the difference is less than 1.5%. All other sequences underestimate *T*_2_ and have poor *T*_2_ accuracy above 95 ms, exceeding 10% and becoming worse with increasing *T*_2_. For example, FatSatMRF_10 underestimates MRF by 2%–6% for *T*_2_ values up to 73 ms. A similar decreasing trend is seen across all versions of SatMRF with a saturation frequency of 10 TRs, with SpaSatMRF_10 performing slightly better than the other options.

### 4.3 In Vivo Experiments

**FIGURE 8:**
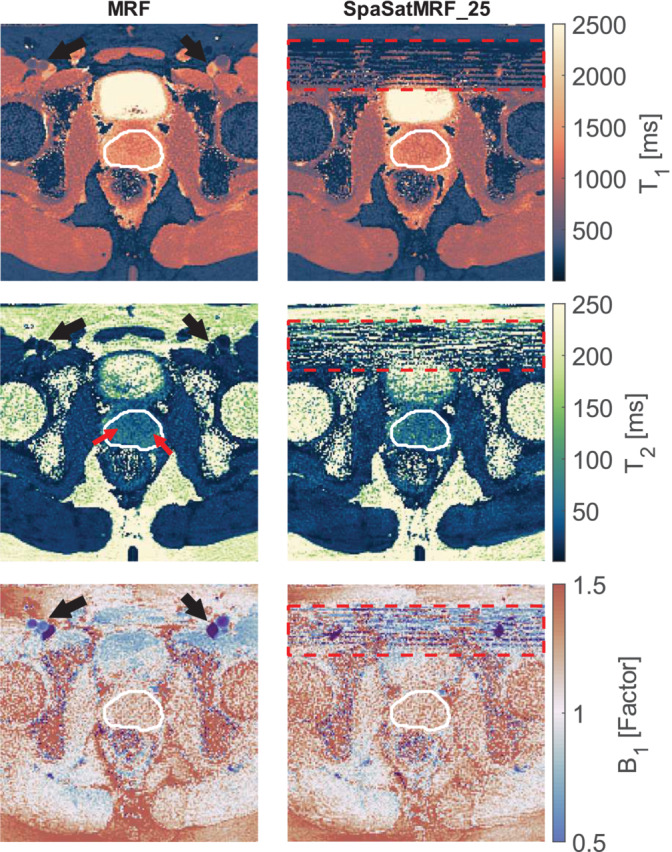
Reconstructed *B*_1_^+^, *T*_1_ and *T*_2_ parameter maps from MRF and SpaSatMRF_25. A single transversal slice of a volunteer scan from separate acquisitions. The femoral blood vessels are shown with black arrows and the prostate is outlined in white. Streak artefacts are clearly visible in all MRF maps, highlighted in the prostate *T*_2_ map by red arrows. In SpaSatMRF_25 reconstructions, the red dashed box highlights the spatially saturated region, and the streaks artefacts from the vessels are well reduced. FatSatMRF_10 maps are provided in Figure S3.

Based on these findings in the phantom experiments, we proceed to in vivo evaluation with the SpaSatMRF_25 sequence as a primary focus although we will later present in vivo data from all SatMRF sequences. The aim was to determine the extent of streak‐like artefact reduction using SatMRF. The streak artefacts are clearly visible in all MRF maps and are most pronounced in the bladder, femoral head, muscle surrounding the femoral vessels and the prostate (Figure 8). Streak‐like artefacts in the *B*_1_^+^ maps were reduced, if not entirely eliminated. Crucially, the streaks visible in the prostate in the *T*_2_ MRF map were also reduced (a zoomed view of the *T*_2_ maps is provided in Figure S2). However, both *T*_1_ and *T*_2_ maps from SatMRF appear noisier than those obtained with the unmodified MRF sequence.

**FIGURE 9:**
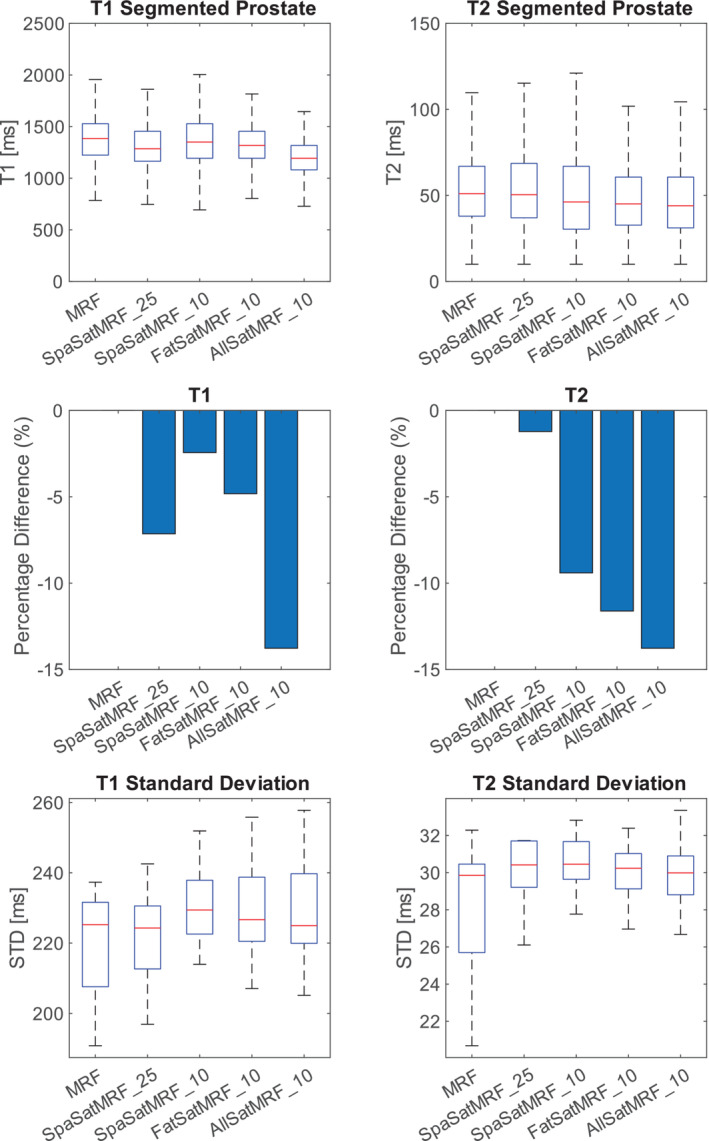
Box plots of prostate segmentations from five asymptomatic volunteers using MRF and SatMRF techniques. *T*_1_ and *T*_2_ boxplots report data from all voxels in all subjects. Standard deviation is determined for each subject.

Figure 9 compares the distribution of *T*_1_ and *T*_2_ values of the segmented prostate from volunteer images using all versions of MRF and SatMRF scans. Subject‐wise boxplot results are given in Figure S4. As in the multicompartment phantom results, we see that *T*_1_ measurement is reduced in SatMRF methods. The median *T*_2_ for SpaSatMRF is within 2% of the MRF measurement. We see a greater difference for the other SatMRF sequences with up to 14% reduction in median *T*_2_ measurement. As a general trend, we note that the standard deviation within each prostate map is increased when applying saturated MRF techniques.

## 5 Discussion

We have demonstrated how chemical and spatial saturation modules can be incorporated into MR Fingerprinting sequences. As highlighted by our flow phantom experiment and an in vivo demonstration, the use of saturation modules in MRF can be used to mitigate artefacts from blood flow in large vessels. We also demonstrated a proof of concept for fat saturation in MRF using our multicompartment phantom.

Analysis of multicompartment phantom data revealed that *T*_1_ measurements obtained with the SpaSatMRF_25 sequence exhibit good alignment with conventional MRF data. The SpaSatMRF_10 implementation had the worst performance. Incidentally, this implementation required high gradient amplitudes within the saturation block. It is possible that diffusion effects played a role in these measurements. Interestingly, the frequency and the duration of the saturation block appear to have little impact on the *T*_1_ estimation.

Regarding *T*_2_ measurements in the multicompartment phantom, SpaSatMRF_25 achieved acceptable accuracy within the range of typical prostate tissue values (4% up to 95 ms) and therefore has potential in prostate mapping. However, it should be noted that the introduction of saturation modules generally has a negative impact on the measurement accuracy of long *T*_2_ components. It appears that the frequency of interruption, rather than the duration, has the strongest impact on *T*_2_ accuracy. In the SpaSatMRF_10 sequence for example, the chain of excitation and readouts was interrupted more frequently than in SpaSatMRF_25, resulting in *T*_2_ errors over 5% at values over 50 ms. We hypothesise that this loss of *T*_2_ estimation may be a result of creating fewer coherence pathways and increasing the time to refocusing. This might be evaluated further by varying the frequency and duration of saturation and evaluating the effects. As we shall explore later, our EPG simulations show that the method is robust against *B*_0_ inhomogeneities, which may otherwise have affected *T*_2_ measurement.

Further to experimental results, we calculated the CRLB for each of the sequences discussed in this paper. This approach has been used before to optimise other MRF sequences [13, 54]. Our analysis found that CRLB values were generally higher for SatMRF sequences, indicating a reduction in encoding power for *T*_1_ and *T*_2_, a finding mirrored in our experimental results. Looking at the simulated signal evolution, we observed signal spikes directly after each saturation module (Figure S5). These signal spikes may have contributed to the diminished encoding power. One might be able to avoid these spikes, by reducing the flip angle of the first pulse following each saturation module. In general, future work could utilise the CRLB to jointly optimise the flip angle train and saturation frequency with the aim to restore the measurement accuracy at long *T*_2_.

In our in vivo study, we analysed the results from segmented prostate maps obtained using SatMRF. We noted a decrease in measured *T*_1_ values when applying both fat and spatial saturation. We hypothesise that magnetisation transfer (MT) effects might explain this discrepancy from the multicompartment phantom observations, as the energy from the saturation pulse also effects the solid pool, leading to reduced signal preservation. This might be further evaluated with more sophisticated simulation tools such as EGP‐X [55] or by incorporating MT parameters into the MRF dictionary as in Hilbert et al. [14].

One crucial aspect of the SatMRF pulse sequence design was the inclusion of an additional spoiler gradient in the saturation module. This decision was motivated by the presence of field inhomogeneities (*dB*_0_), which effectively produce a small but always‐on gradient. This phenomenon leads to the accumulation of phase over time, which must be addressed in order to produce accurate tissue maps. We therefore utilised integer scaled spoiling gradients, proportional to the duration of the saturation block. The EPG formulism was instrumental in illustrating the process and emphasising the potential consequences of incorrectly calculated spoiling. By incorporating this additional spoiler gradient, we were able to mitigate the effects of *B*_0_ field inhomogeneities and ensure accurate measurements in the SatMRF sequences.

There are several alternative approaches to saturation in MRF. Firstly, one might consider implementing sophisticated RF pulses, such as the simultaneous saturation excitation pulse (SatEx) [56], into an MRF paradigm. This novel RF pulse design simultaneously excites a slice and suppresses the adjected regions, enabling parallel saturation. Incorporating this technique into MRF could mitigate artefacts from inflow effects.

Regarding fat, another approach to consider would be mapping the fat content as opposed to suppressing fat signals. Techniques such as those described by Cencini et al. and Ostenson et al. might be combined with spatial SatMRF [16, 17]. For example, fat saturation pulses could be included sporadically allowing unique signal evolution to form for different fat water combinations. It may then be possible to quantify the fat water fraction using a dedicated dictionary covering a range of percentage fat/water fractions. This would have the benefit of providing additional information, which may improve tissue characterisation, but it would not necessarily address artefacts originating from fat tissue.

Besides prospective techniques, it may also be possible to mitigate flow and bright fat signal‐related artefacts retrospectively. Sørland et al. recently reported an automated post‐processing approach to streak artefact reduction in MRF, as an alternative to the pulse sequence modification set out in this study [57]. This method utilised Region‐Optimised Virtual (ROVir) [58] coils applied to MRF k‐space data, enhancing the signal from a region of interest while suppressing signal from sources of interference or artefacts. Less than 5% of the signal energy in the femoral vessels was retained, visibly reducing the streak‐like artefacts. The method did not change the sensitivity of the MRF sequence but did lead to sacrifices in the signal energy in the prostate region of interest.

Moving towards considerations in clinical implementation, it should be noted that incorporating saturation modules into the MRF sequence increases the overall acquisition time. The SpaSatMRF_25 sequence is 6% longer in duration than the original MRF sequence, whereas the AllSatMRF_10 sequence increases the total scan time by 32%. Moreover, accurate placement of the spatial saturation bands required a dedicated vessel scout scan (1 min and 22 s). The workflow can be simplified by placing the spatial saturation bands parallel above and below the imaging slice. However, fast‐flowing blood may still enter the imaging slice, unless the saturation module is repeated very frequently. The aforementioned SatEx pulse design might offer an elegant solution to this problem. However, SatEx is yet to be demonstrated in the context of FISP‐based sequences and MRF and would not be applicable to fat or chemical saturation.

In conclusion, our work demonstrates how to incorporate saturation techniques into MRF sequences. Phantom experiments validate that *T*_1_ accuracy is retained and *T*_2_ accuracy is acceptable up to 95 ms. We used prostate imaging as an example of flow artefact reduction in vivo. The accuracy of *T*_1_ and *T*_2_ maps within the typical prostate range was retained.

## Supporting information


**Figure S1** Pulse Sequence Diagrams and Extended Phase Graphs for each example sequence described in Figure 1.
**Figure S2.** Zoomed View (2x) of T2 Maps from an asymptomatic volunteer. Streak artefacts are visible in the prostate (white outline) and are reduced in the SatMRF Map.
**Figure S3.** In Vivo Example with FatSatMRF_10 sequence.
**Figure S4.** T1 and T2 values from whole prostate segmentation of MRF and SatMRF Maps for five asymptomatic volunteers. All data is combined in the white boxplot for each sequence.
**Figure S5.** Left MRF, Right SatMRF. Dictionary Profiles for a constant T1, T2 and B1 value.

## Data Availability

The data that support the findings will be available upon request from the corresponding author.
