# Supplementary material for: Incorporating Spatial and Spectral Saturation Modules Into MR Fingerprinting
Source: NMR Biomed. 2025 Jan 25;38(3):e70000. doi: 10.1002/nbm.70000 (PMC11771585; doi:10.1002/nbm.70000)
Supplement: Supplementary file 1 — Figure S1 Pulse Sequence Diagrams and Extended Phase Graphs for each example sequence described in Figure 1. Figure S2. Zoomed View (2x) of T2 Maps from an asymptomatic volunteer. Streak artefacts are visible in the prostate (white outline) and are reduced in the SatMRF Map. Figure S3. In Vivo Example with FatSatMRF_10 sequence. Figure S4. T1 and T2 values from whole prostate segmentation of MRF and SatMRF Maps for five asymptomatic volunteers. All data is combined in the white boxplot for each sequence. Figure S5. Left MRF, Right SatMRF. Dictionary Profiles for a constant T1, T2 and B1 value. [file NBM-38-e70000-s001.docx]

Supplementary Material


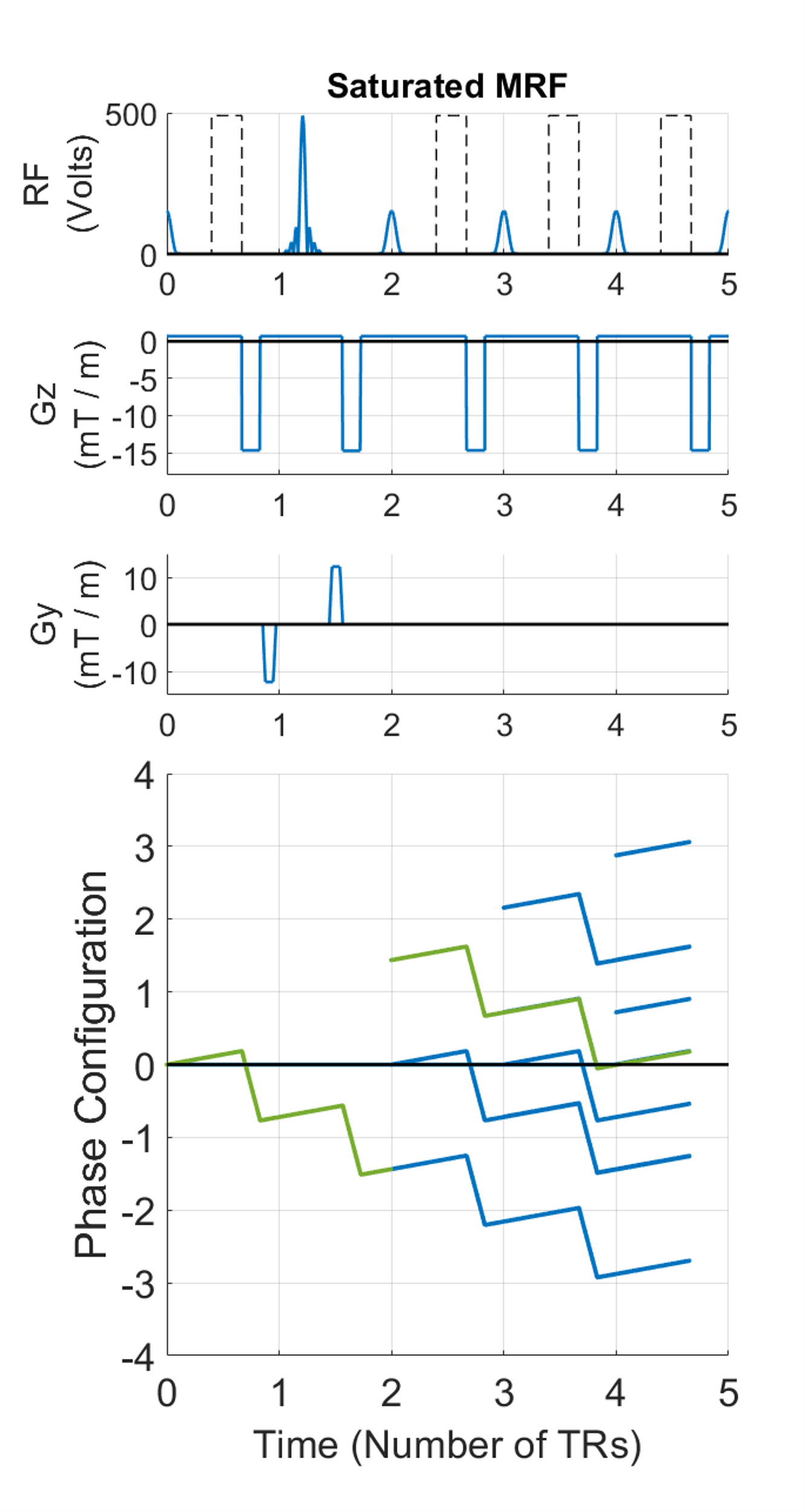

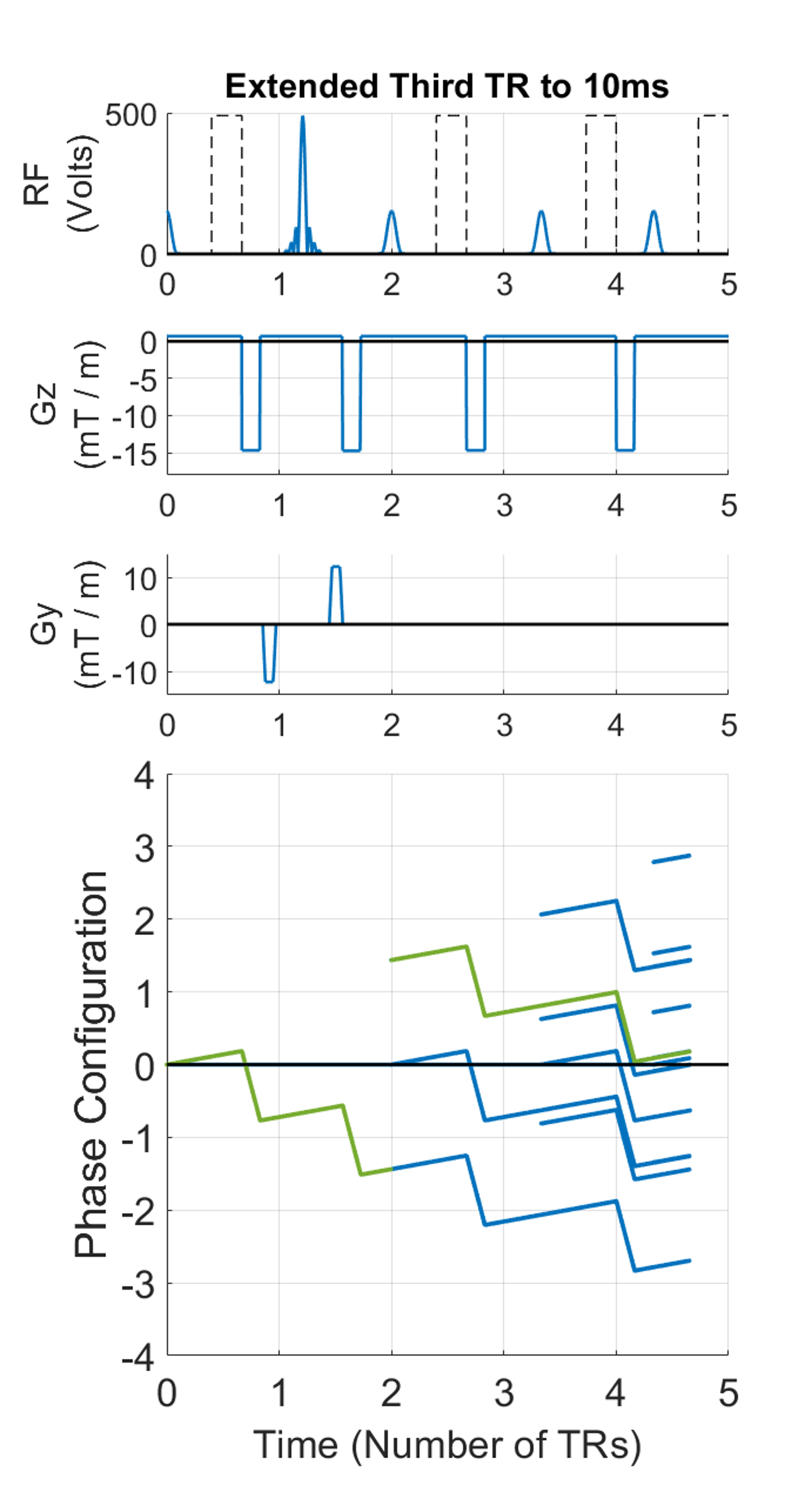

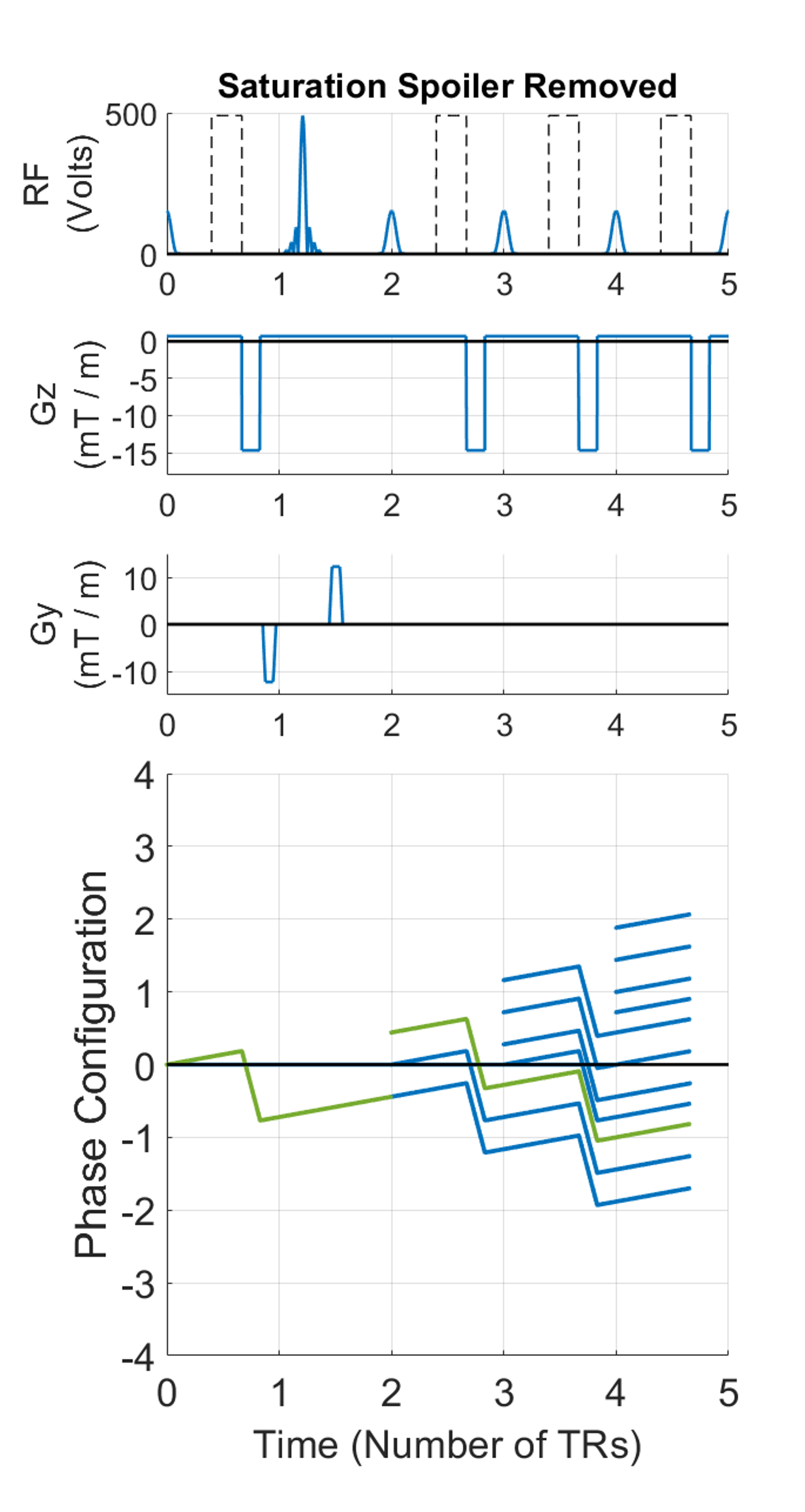


Supplementary Figure 1) Pulse Sequence Diagrams and Extended Phase Graphs for each example sequence described in Figure 1.

*
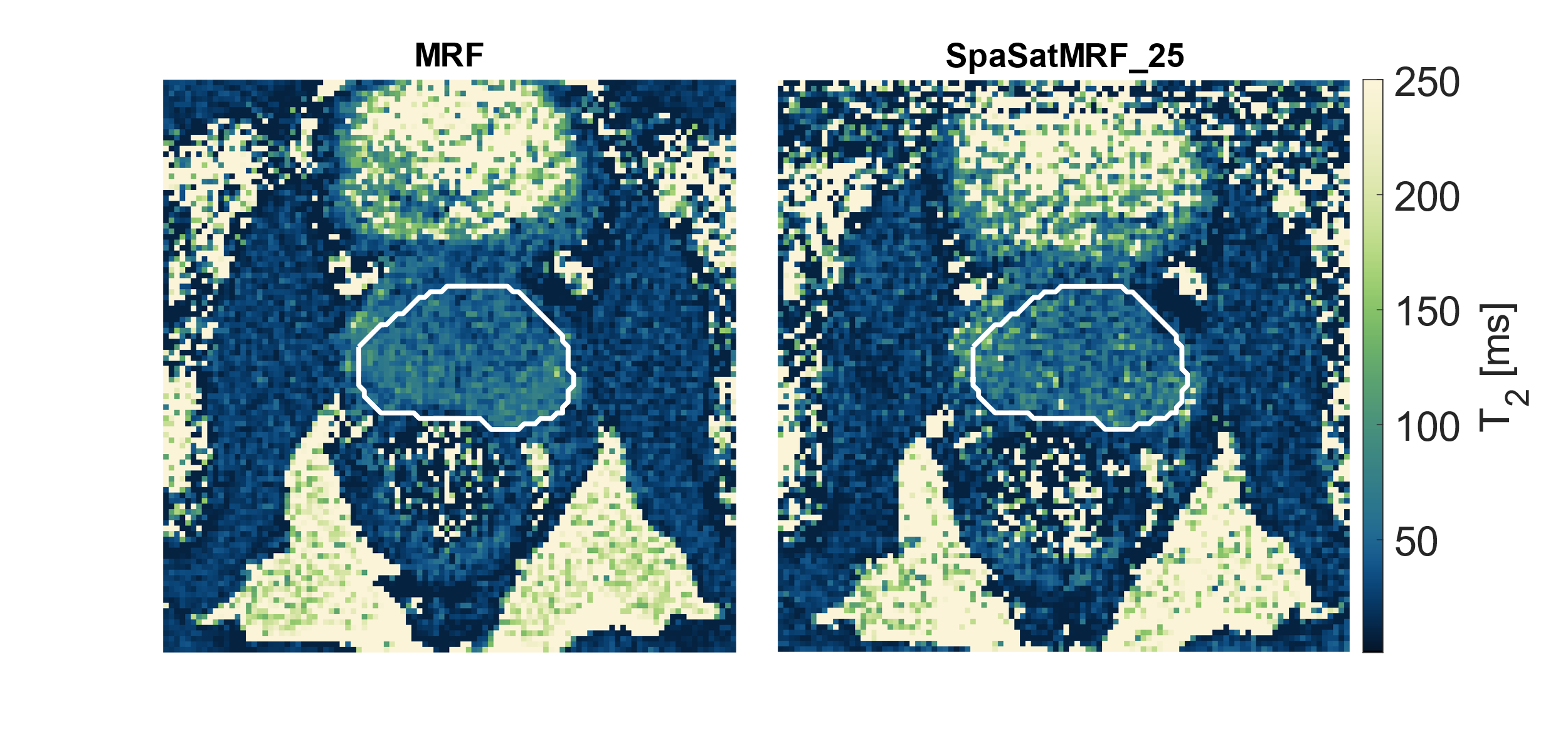
*

Supplementary Figure 2) Zoomed View (2x) of T2 Maps from an asymptomatic volunteer. Streak artefacts are visible in the prostate (white outline) and are reduced in the SatMRF Map.


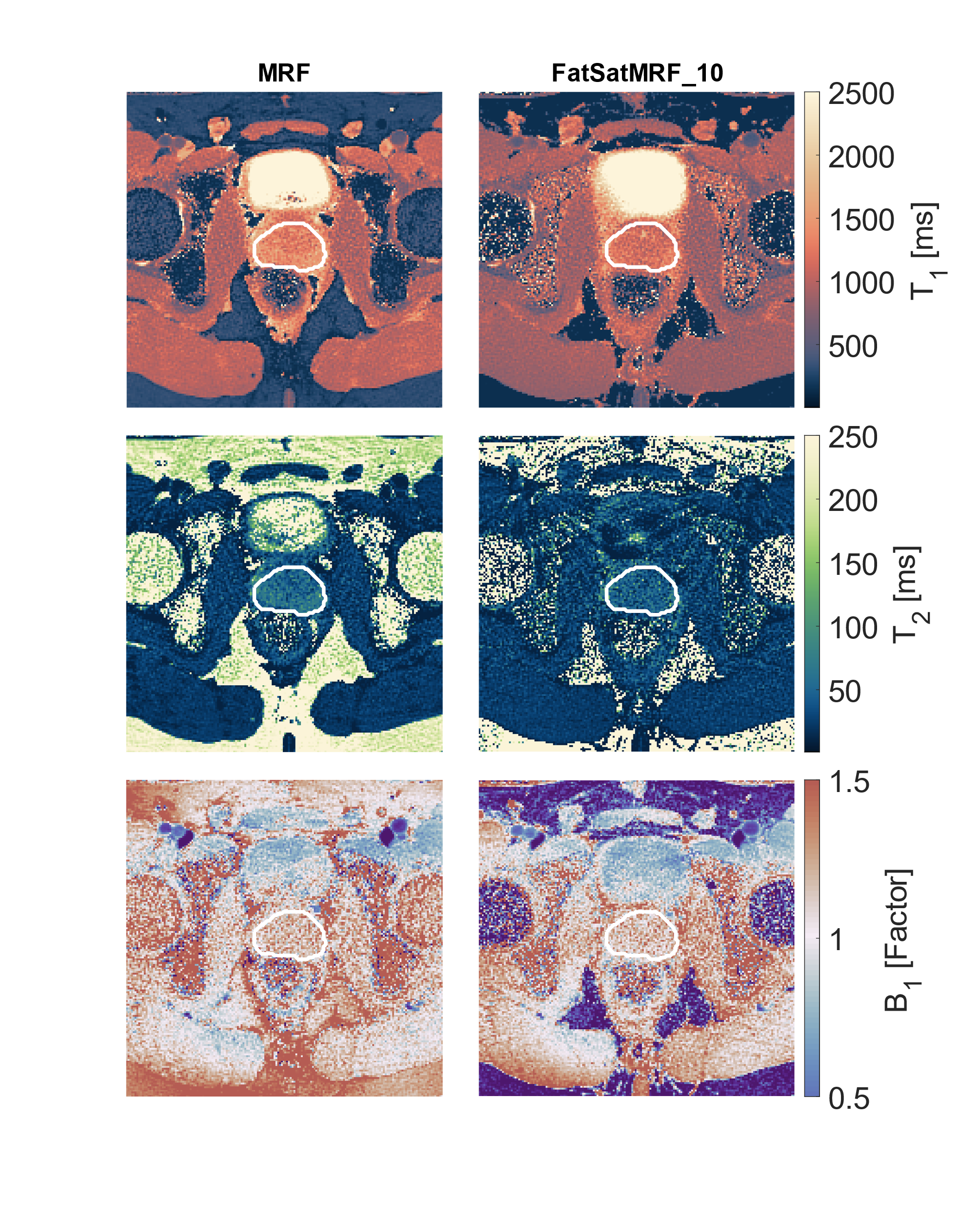


Supplementary Figure 3) In Vivo Example with FatSatMRF_10 sequence.

*
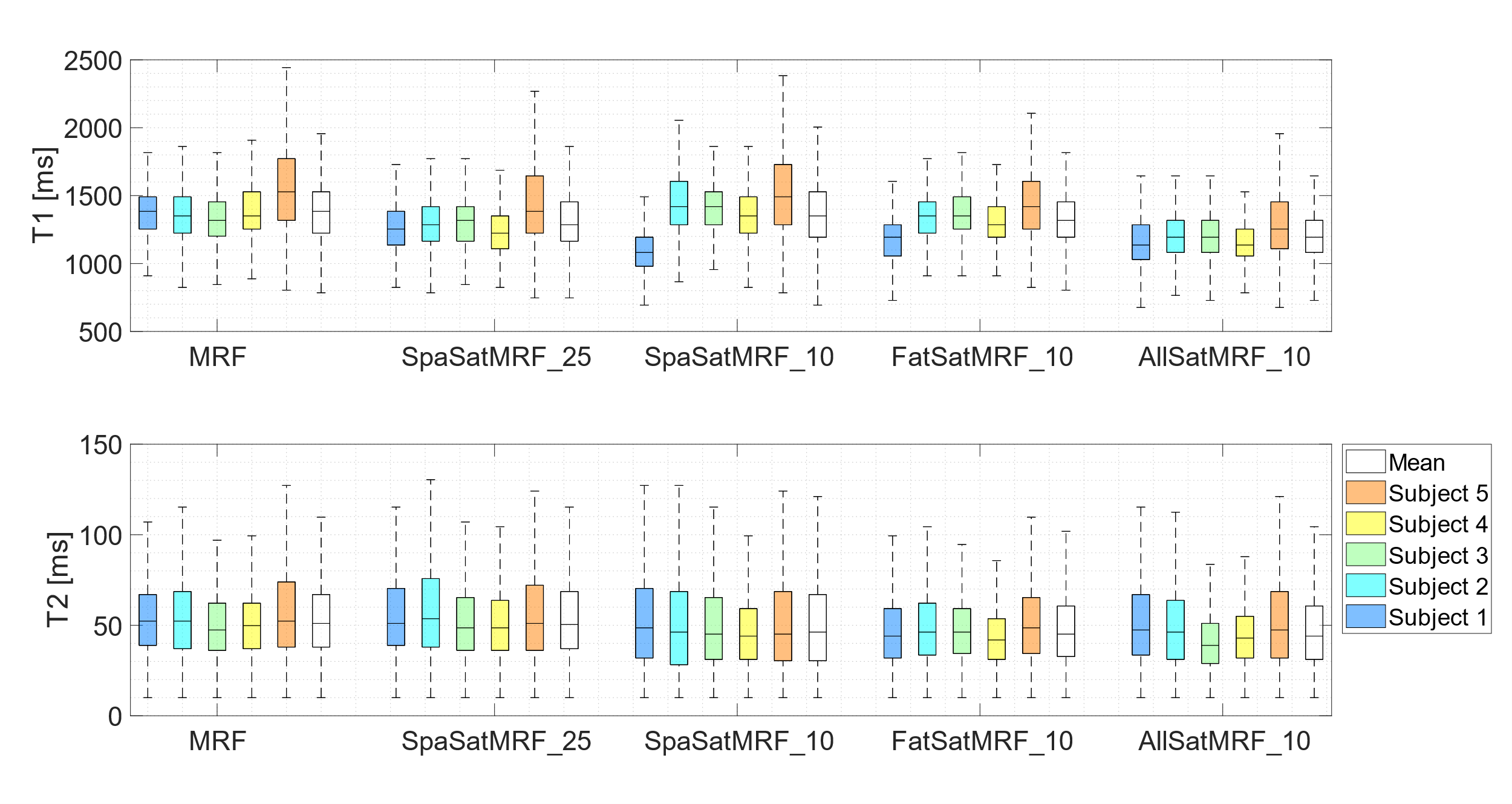
*

Supplementary Figure 4) T1 and T2 values from whole prostate segmentation of MRF and SatMRF Maps for five asymptomatic volunteers. All data is combined in the white boxplot for each sequence.


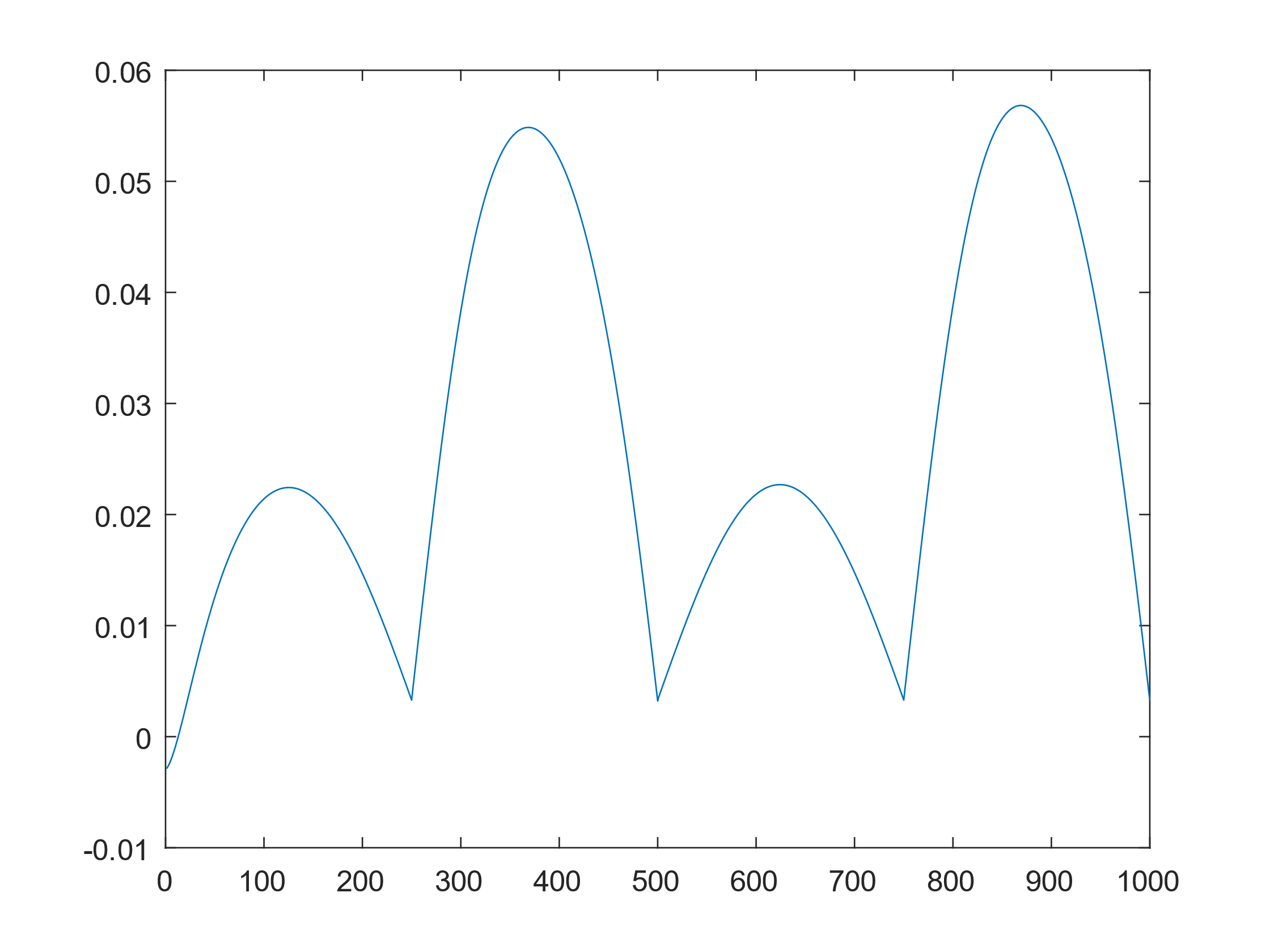

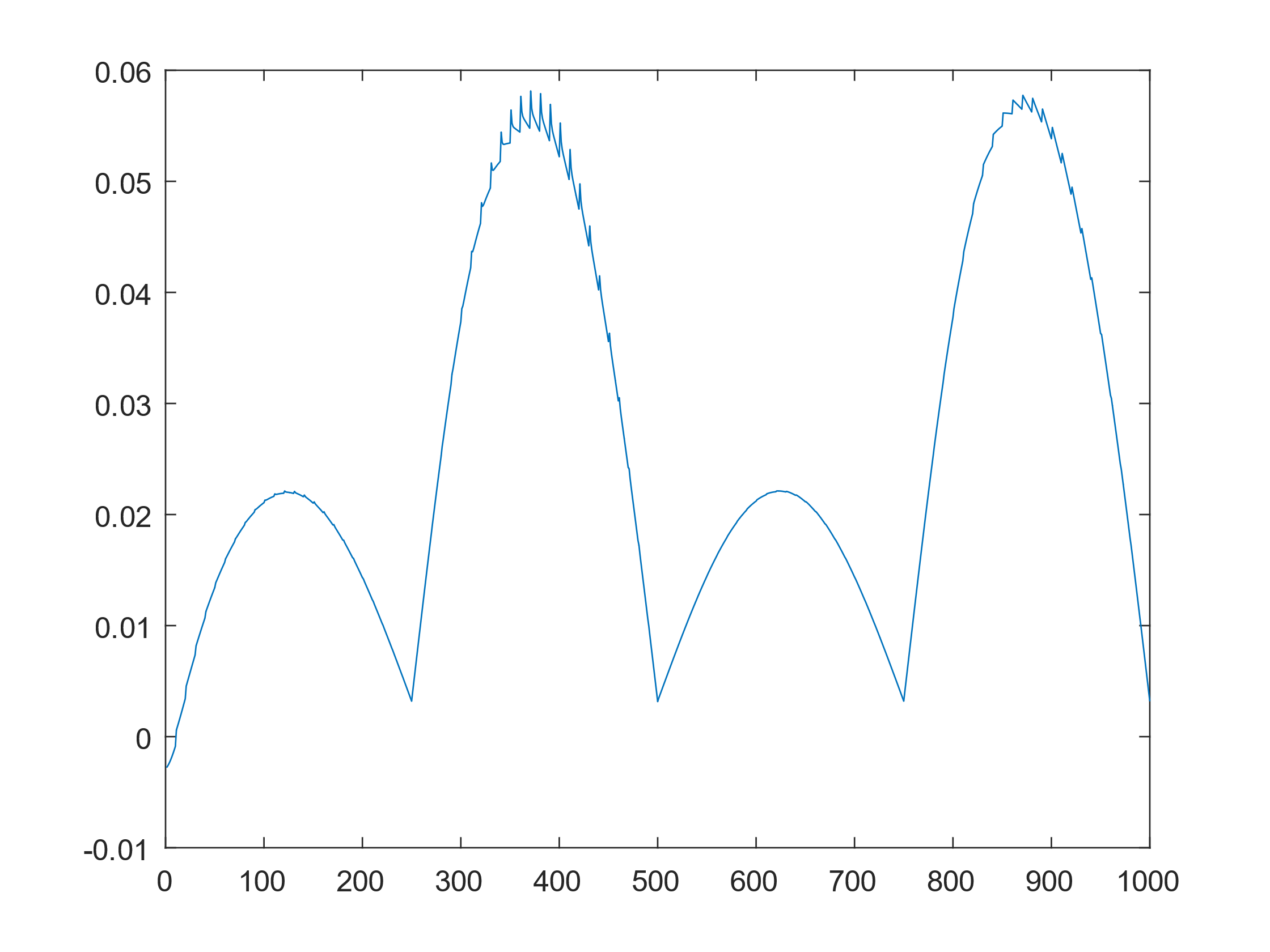


Supplementary Figure 5) Left MRF, Right SatMRF. Dictionary Profiles for a constant T1, T2 and B1 value.
